# Supplementary material for: Transcriptome Analysis of Durusdinium Associated with the Transition from Free-Living to Symbiotic
Source: Microorganisms. 2021 Jul 22;9(8):1560. doi: 10.3390/microorganisms9081560 (PMC8399598; doi:10.3390/microorganisms9081560)

Supplementary Information
Transcriptome analysis of *Durusdinium* associated with the transition from free-living to symbiotic

Ikuko Yuyama, Naoto Ukawa and Tetsuo Hashimoto

Tale of Content

I. SI Materials and Methods
I1. Samples……………………………………………………………………………………..2
I2. De-novo assembly of the Symbiodiniaceae derived sequence dataset……………………..2
I3. Detection of differentially expressed genes…………………………………………..…..2
I4. Phylogenetic analysis of dapdiamide A synthase………………………………………..2,4

II. Supplementary Figures
Fig. S1. Number of differentially expressed genes (DEGs) identified by comparing non-symbiotic and symbiotic *Durusdinium* using edgeR method and Student's *t*-test with bootstrap resampling data…………………………………………………………………………………5
Fig. S2. The results of GO (gene ontology) analysis using down-regulated 1,025 DEG and up-regulated 189 DEG against all *Durusdinium* mRNA sequence isolated our studies…………..6

I.SI Materials and Methods

I.1. Samples

Symbiodiniaceae strains CCMP 2556 (D-type, genus *Durusdinium*) were purchased from the Bigelow Laboratory for Ocean Sciences (West Boothbay Harbor, ME, USA; https://ccmp.bigelow.org/). Cultures were grown at 24°C under a 12-h/12-h light/dark cycle at 80 µmol m^{-2} s^{-1}, in 100 mL filtered seawater in Erlenmeyer flasks. Two biological replicates were prepared and grown separately for 3 weeks.

I.2. RNA isolation, and transcriptome sequencing

Algal cells were collected by centrifuging from 100 mL cultures, and cells were frozen in liquid nitrogen and crushed in a mortar with a pestle. Total RNA was extracted using TRIzol reagent and Phasemaker tubes (Invitrogen, Carlsbad, CA, USA), and contaminating DNA was eliminated using the TURBO DNA-free kit (Invitrogen). The cDNA library was generated using the Kapa stranded mRNA-seq kit (Kapa Biosystems Inc., Wilmington, MA, USA) and a FastGene adapter kit (FastGene, Tokyo, Japan). Sequencing was performed using an Illumina NextSeq500 system (Illumina, San Diego, CA, USA) at a read length of 151 bp. Library preparation and paired-end sequencing were conducted by Bioengineering Laboratories Co., Ltd. (Kanagawa, Japan).

I.3. De-novo assembly of the Symbiodiniaceae derived sequence dataset

RNA-seq reads were quality filtered using Fastp (v0.12.6). The trimmed reads data are available in the DNA Data Bank of Japan (DDBJ) Sequenced Read Archive (accession No. DRA010343). All reads were used in de novo transcript assembly using Trinity (v2.5.1). Among generating 191,008 contigs, sequences containing open reading frames (OFRs) were isolated using Transdecoder (v5.3.0). Symbiodiniaceae derived contigs were determined by a BLASTN search ($E \leq 1 \times 10^{-15}$) against Symbiodiniaceae transcript data of Ladner et al. (2012), Shoguchi et al. (2013, 2018), and Lin et al. (2015) [13,15,16,23]. Algal-derived contigs were subjected to the CD-hit program (v3.2.3) to remove the redundancies (identity > 95%). A total number of 25,068 contigs were finally obtained.

I.4. Detection of differentially expressed genes

Illumina HiSeq2000 transcriptome data for symbiotic state of *Durusdinium trenchii* were obtained from the DDBJ Sequence Read Archive (accession nos. DRR119964-119967). Data obtained at 10th and 20 days after coral incubation with *D. trenchii* [10], and those obtained in the present study were used for DEG analysis. Quality filtered reads of *D. trenchii* in non-symbiotic and symbiotic states were mapped onto the obtained algal derived contigs obtained using Bowtie2 (v3.2.3) [24]. The mapping results were analyzed using the eXpress program (v1.5.1) [25] to calculate expression levels, which were compared between the non-symbiotic and symbiotic states using the *TCC* package (v1.10.0) of R (v3.2.3) [26]; the edgeR method was used to determine expression variation, using the setting test method = edgeR in the estimate DE function, a false discover rate (FDR) < 0.05, and $\log_2$(fold change) > 1.

To confirm the calculated RNA-Seq results, we performed bootstrap resampling of the raw read data, with 100 replicates per sample using the isoDE2 package [27], with 100 replicates (n = 100) for each free-living or symbiotic sample (duplicate) to examine the expression changes between these states. Since the bootstrap resampling results showed considerable variation among replicates for the data collected at day 10 of symbiosis, we eliminated these data from our analysis to detect changes related to symbiosis. T-test using 100 resampling result in generate undetectable lower p-values of most genes, so 10 data was randomly selected from 100 resampling data and t-test was performed. Differences in the mean (among 100 replicates) expression of each gene between the two states were detected using Student's *t*-test (P < 0.025). The P value was set to correspond to the number of DEGs detected in edgeR analysis (q = 0.01). These obtained DEGs were compared with those detected by the *TCC* analysis, and those in common were selected. We also selected genes with an expression change of $\log_2$(fold change) > 1 between states. To

estimate the function of each DEG, we performed a BLASTx search using the BLAST+ program (v2.2.31+) against the UniProtKB and Swiss-Prot databases with a cut-off of E ≤ 1 × 10^{-4}. Enrichment analysis was performed using DAVID software (v6.8)[28]. As a background dataset for this analysis, we used the annotation results of all *Durusdinium* sequences isolated in this study. We focused on the gene ontology (GO) term "biological process category" and confirmed significantly enriched GO terms (P < 0.05) among all up- and downregulated DEGs. The enriched GO terms were visualized as a tree map plot using the ReviGO [29]. Genes associated with the enriched GO term were extracted and their expression levels reconfirmed and visualized as a heatmap (Figure S1). Normalized FPKM values of the DEGs were converted to Z scores and plotted using the heatmap.2 function in R.

I.5. Phylogenetic analysis of dapdiamide A synthase

Among the DEGs, we focused on two dapdiamide A (antibiotic) synthetase genes that are characteristic of *Dursdinium*. A phylogenetic analysis of dapdiamide A synthase was performed using the homologous gene sequences from diverse organisms to confirm that the genes were derived from *Durusdinium* rather than from bacteria. Two isoforms of dapdiamide A synthase (TRINITY_DN38519_c0_g1_i5.p1 and TRINITY_DN38519_c0_g1_i1.p1) were isolated from *Durusdinium* and used to isolate homologues via a BLASTp search against the National Center for Biotechnology Information (NCBI) non redundant database. We retrieved 1,000 sequence hits using a cut-off of E ≤ 1 × 10^{-34}. The resulting sequences were derived from eukaryotes, prokaryotes, and archaeans. For each gene set, the CD-HIT program (v.4.7) was used to cluster nucleotide sequences of ≧ 98% similarity, and retained a representative sequence from each cluster, resulting in 104 (+11) eukaryotic sequences including 11 from Symbiodiniaceae and 475 eubacterial sequences. To further reduce the number of eubacterial sequences used for phylogenetic analysis, sequences were extracted every 20 columns from the list of eubacterial sequences, to obtain a total of 23 sequences. The top 10 eubacterial sequence hits in the first BLASTp search were added to create a list of eubacterial sequences. We also included 3 archaeal sequences, 11 Symbiodiniaceae genes that were also included in the first BLASTp search result, and 2 *Durusdinium* genes isolated from the present study to create a dataset of 142 sequences for phylogenetic analysis. The sequences were then aligned using the MAFFT (v.7.475)[30]. Ambiguous and poorly aligned sites were removed using the TrimAI program (v.1.4, rev15) with the "-gappyout" option.[31]. We removed 25 sequences containing many ambiguous sites from the alignment results, and finally obtained 117 sequences with 318 amino acids for phylogenetic analysis. IQ-Tree software (v.1.5.5) was used to infer the maximum likelihood (ML) tree in ultrafast bootstrap analysis with 100 replicates.

References

1. Shoguchi, E.; Shinzato, C.; Kawashima, T.; Gyoja, F.; Mungpakdee, S.; Koyanagi, R.; Takeuchi, T.; Hisata, K.; Tanaka, M.; Fujiwara, M.; et al. Draft Assembly of the Symbiodinium Minutum Nuclear Genome Reveals Dinoflagellate Gene Structure. *Current Biology* **2013**, *23*, 1399–1408, doi:10.1016/j.cub.2013.05.062.
2. Lin, S.; Cheng, S.; Song, B.; Zhong, X.; Lin, X.; Li, W.; Li, L.; Zhang, Y.; Zhang, H.; Ji, Z.; et al. The Symbiodinium Kawagutii Genome Illuminates Dinoflagellate Gene Expression and Coral Symbiosis. *Science* **2015**, *350*, 691–694, doi:10.1126/science.aad0408.
3. Shoguchi, E.; Beedessee, G.; Tada, I.; Hisata, K.; Kawashima, T.; Takeuchi, T.; Arakaki, N.; Fujie, M.; Koyanagi, R.; Roy, M.C.; et al. Two Divergent Symbiodinium Genomes Reveal Conservation of a Gene Cluster for Sunscreen Biosynthesis and Recently Lost Genes. *BMC Genomics* **2018**, *19*, 458, doi:10.1186/s12864-018-4857-9.

4. Ladner, J.T.; Barshis, D.J.; Palumbi, S.R. Protein Evolution in Two Co-Occurring Types of Symbiodinium: An Exploration into the Genetic Basis of Thermal Tolerance in Symbiodinium Clade D. *BMC Evol. Biol.* **2012**, *12*, 217, doi:10.1186/1471-2148-12-217.
5. Yuyama, I.; Ishikawa, M.; Nozawa, M.; Yoshida, M.; Ikeo, K. Transcriptomic Changes with Increasing Algal Symbiont Reveal the Detailed Process Underlying Establishment of Coral-Algal Symbiosis. *Sci Rep* **2018**, *8*, 16802, doi:10.1038/s41598-018-34575-5.
6. Langmead, B.; Salzberg, S.L. Fast Gapped-Read Alignment with Bowtie 2. *Nat Methods* **2012**, *9*, 357–359, doi:10.1038/nmeth.1923.
7. Roberts, A.; Pachter, L. Streaming Fragment Assignment for Real-Time Analysis of Sequencing Experiments. *Nat Methods* **2013**, *10*, 71–73, doi:10.1038/nmeth.2251.
8. Sun, J.; Nishiyama, T.; Shimizu, K.; Kadota, K. TCC: An R Package for Comparing Tag Count Data with Robust Normalization Strategies. *BMC Bioinformatics* **2013**, *14*, 219, doi:10.1186/1471-2105-14-219.
9. Al Seesi, S.; Tiagueu, Y.T.; Zelikovsky, A.; Măndoiu, I.I. Bootstrap-Based Differential Gene Expression Analysis for RNA-Seq Data with and without Replicates. *BMC Genomics* **2014**, *15*, S2, doi:10.1186/1471-2164-15-S8-S2.
10. Huang, D.W.; Sherman, B.T.; Zheng, X.; Yang, J.; Imamichi, T.; Stephens, R.; Lempicki, R.A. Extracting Biological Meaning from Large Gene Lists with DAVID. *Curr Protoc Bioinformatics* **2009**, *Chapter 13*, Unit 13.11, doi:10.1002/0471250953.bi1311s27.
11. Supek, F.; Bošnjak, M.; Škunca, N.; Šmuc, T. REVIGO Summarizes and Visualizes Long Lists of Gene Ontology Terms. *PLOS ONE* **2011**, *6*, e21800, doi:10.1371/journal.pone.0021800.
12. Katoh, K.; Misawa, K.; Kuma, K.; Miyata, T. MAFFT: A Novel Method for Rapid Multiple Sequence Alignment Based on Fast Fourier Transform. *Nucleic Acids Research* **2002**, *30*, 3059–3066, doi:10.1093/nar/gkf436.
13. Capella-Gutiérrez, S.; Silla-Martínez, J.M.; Gabaldón, T. TrimAl: A Tool for Automated Alignment Trimming in Large-Scale Phylogenetic Analyses. *Bioinformatics* **2009**, *25*, 1972–1973, doi:10.1093/bioinformatics/btp348.

Supplementary Figure S1

Number of differentially expressed genes (DEGs) identified by comparing non-symbiotic and symbiotic *Durusdinium* using edgeR method and Student's *t*-test with bootstrap resampling data. A total of 4,587 DEGs were common between both analyses and 1,214 genes had $\log_2$(fold change) > 1 between the two states. Among the 1,214 genes, 189 were upregulated and 1,025 were downregulated during endosymbiosis establishment.

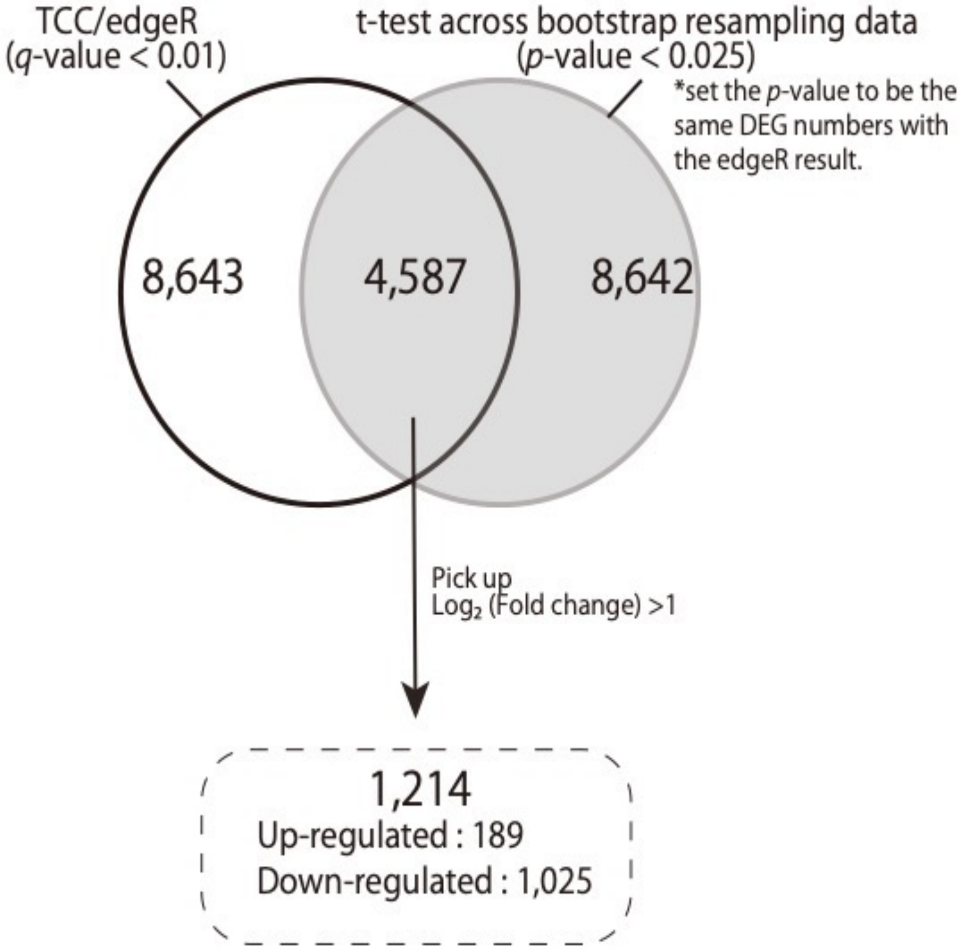

Supplementary Figure S2

The results of GO (gene ontology) analysis using down-regulated 1,025 DEG and up-regulated 189 DEG against all *Durusdinium* mRNA sequence isolated our studies. The top enriched GO terms for upregulated genes included photosynthetic electron transport in photosystem II, positive regulation of GTPase activity, protein–chromophore linkage, and photosynthesis. Seven processes were related to downregulated DEGs, including microtubule-based processes, small GTPase-mediated signal transduction, mRNA splicing via spliceosome, anion transport, peptidyl-cysteine S-trans-nitrosylation, chaperone-mediated protein folding, and protein folding.

Up-regulated in Day 20 symbiosis

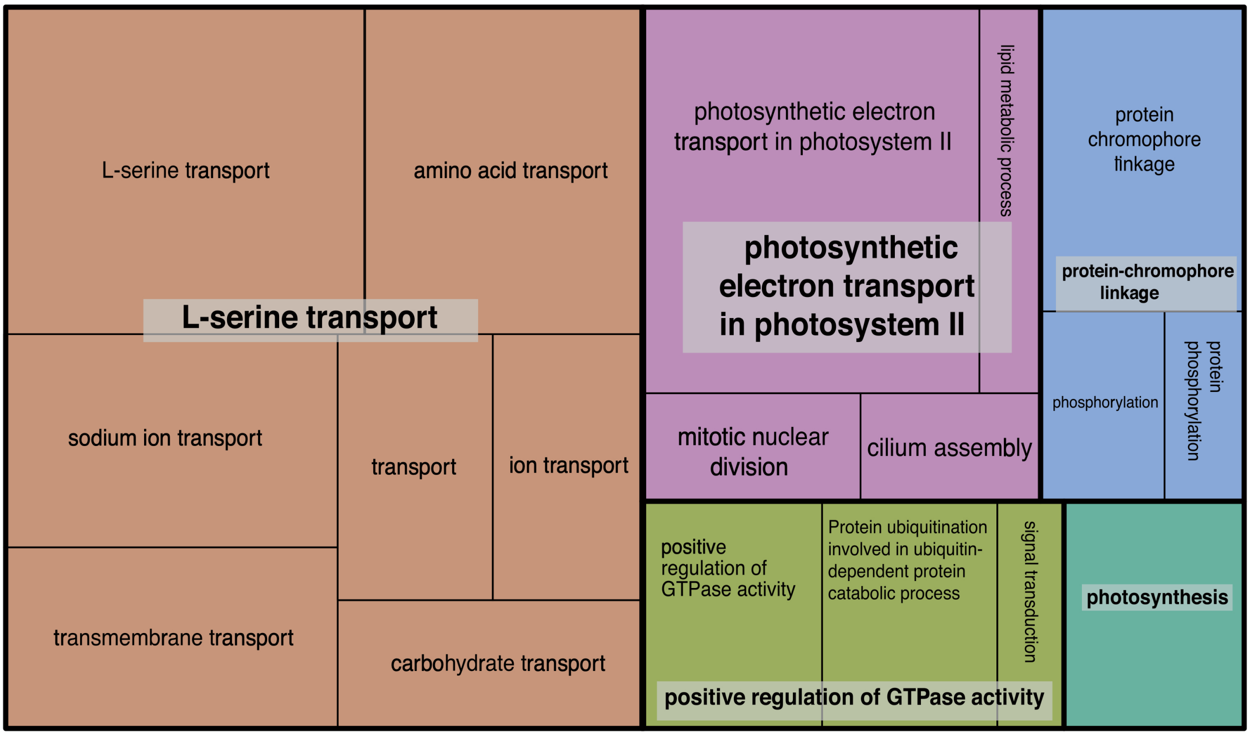

Down-regulated in Day 20 symbiosis

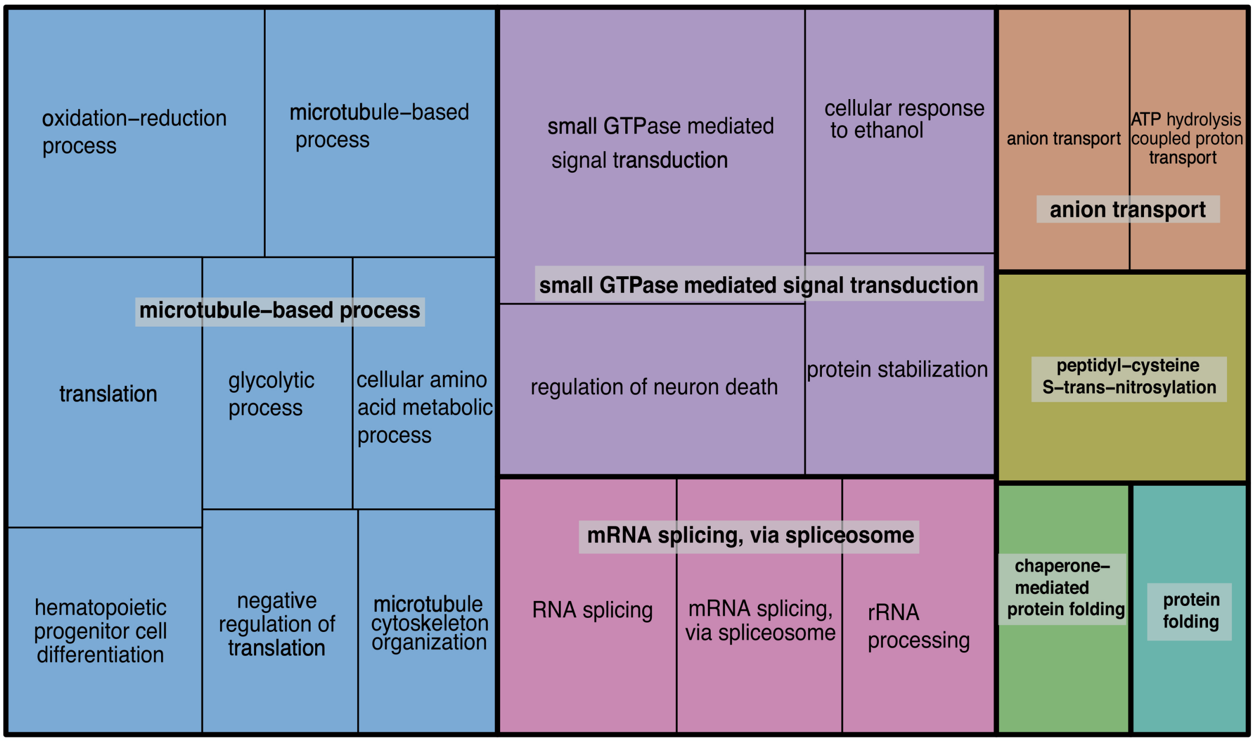

Supplement: Supplementary file 1 [file microorganisms-09-01560-s001.zip › microorganisms-1202611-supplementary.pdf]
